# Use of an embedded, micro-randomised trial to investigate non-compliance in telehealth trials

**DOI:** 10.1186/1745-6215-16-S2-P222

**Published:** 2015-11-16

**Authors:** Lisa Law, James Wason

**Affiliations:** 1 MRC Biostatistics Unit hub for trials methodology research, Cambridge, UK

## 

Telehealth interventions can provide remote monitoring of patients at home, through the use of electronic devices. For example, diabetics are asked to record regular blood glucose levels, and individuals with respiratory illnesses are asked to fill in electronic symptom questionnaires. As clinicians can monitor this ongoing data, they are aware of signs of poor health and can intervene to avoid a serious deterioration. The effectiveness of this type of intervention is dependent on how much data the patient provides, i.e. on patient compliance. Non-compliance is a big problem among telehealth trials. Even though it is often reported in telehealth trials, there is rarely a formal investigation of measures to improve compliance. With the expense of running and planning clinical trials, the focus remains on testing the primary, clinical outcome.

An efficient solution is to investigate non-compliance within the primary trial. We propose an embedded secondary trial to take place within the telehealth arm of a randomised controlled trial. This trial would investigate several factors of the delivery of the telehealth intervention, to see which is the most successful in terms of compliance. For further efficiency we will investigate a new technique called micro-randomisation. This involves randomising patients many times throughout the trial, to the various factors related to compliance. When factors are compared, patients act as their own control, so reducing variability caused by within-patient correlation, as in cross-over trials.

We use simulation studies to demonstrate the validity of our proposed design and its advantages over conventional approaches.

